# LSD1 binds to HPV16 E7 and promotes the epithelial-mesenchymal transition in cervical cancer by demethylating histones at the Vimentin promoter

**DOI:** 10.18632/oncotarget.13516

**Published:** 2016-11-23

**Authors:** Yuan Liu, Yanan Wang, Chunqin Chen, Jiawen Zhang, Wenyan Qian, Yu Dong, Zhiqiang Liu, Xi Zhang, Xiaoyun Wang, Zhenbo Zhang, Xiaobing Shi, Sufang Wu

**Affiliations:** ^1^ Department of Obstetrics and Gynecology, Shanghai General Hospital, Shanghai Jiaotong University, Shanghai, China; ^2^ Department of Obstetrics and Gynecology, Shanghai Tenth People's Hospital, Shanghai Tongji University, Shanghai, China; ^3^ Department of Gynecology and Obstetrics, Kunshan Hospital of Traditional Chinese Medicine Affiliated to Nanjing University of Chinese Medicine, Jiangsu, China; ^4^ Department of Obstetrics and Gynecology, Shanghai Xinhua hospital, Shanghai Jiaotong University, Shanghai, China; ^5^ Division of Cancer Medicine, Department of Lymphoma and Myeloma, Center for Cancer Immunology Research, The University of Texas M.D. Anderson Cancer Center, Houston, TX, USA; ^6^ Department of Physiology and Neurobiology, University of Connecticut, CT, USA; ^7^ Department of Molecular Carcinogenesis and Center for Cancer Epigenetics, The University of Texas MD Anderson Cancer Center, Houston, TX, USA; ^8^ Genes and Development and Molecular Carcinogenesis Graduate Program, The University of Texas Graduate School of Biomedical Sciences, Houston, TX, USA

**Keywords:** LSD1, HPV16E7, cervical cancer, EMT

## Abstract

Lysine-specific demethylase 1 (LSD1), which specifically demethylates histone H3 lysine 4 (H3K4) and lysine 9 (H3K9), is dysregulated in several cancers. We found that ectopic expression of *LSD1* in cervical cancer cells promoted invasion and metastasis *in vitro* and *in vivo*, reduced the expression of the epithelial marker E-cadherin, and induced the expression of the mesenchymal marker, Vimentin. By contrast, LSD1 knockdown had the opposite effect and attenuated the HPV16 E7-induced epithelial-mesenchymal transition (EMT). We proposed a novel mechanism, whereby LSD1 is recruited to the Vimentin promoter and demethylates H3K4me1 and H3K4me2. Notably, HPV16 E7 enhanced the expression of LSD1, formed a complex with LSD1, and suppressed LSD1 demethylase activity by hindering the recruitment of LSD1 to the Vimentin promoter. Thus, LSD1 is a primary and positive regulator of the HPV16 E7-induced EMT and an attractive therapeutic target for alleviating HPV16 E7-induced EMT and tumor metastasis.

## INTRODUCTION

According to the World Health Organization, cervical cancer is one of the most common cancers in women worldwide [1], especially in China and developing countries. There were more than 98,900 new cases of cervical cancer and 30,500 deaths reported in China in 2015 [2]. Human papilloma virus (HPV), a small, circular, double-stranded DNA virus infecting epithelial cells, has been reported to be necessary but not sufficient to induce tumorigenesis in host squamous epithelial cells [3, 4]. Persistent high-risk HPV infection of the cervix is the defining feature of cervical cancer [5]. The major oncogenes, E6 and E7, cooperate to control the cell cycle and promote cell growth by inhibiting the activity of important tumor suppresser proteins, including p53, retinoblastoma (Rb) family members, PDZ domain-containing proteins, etc [6]. E7 overcomes proliferation arrest by sequestering Rb from E2F complexes, disrupting a series of signaling pathways and thus facilitating tumor growth, invasion or metastasis [7, 8]. Despite the clear and complete understanding of these mechanisms, the success of HPV-targeted treatments has not improved.

Prophylactic vaccination against high-risk HPV types 16 and 18 has been used in certain developed countries, like Australia and the United Kingdom. However, for some reason, these vaccines are not applied in developing countries, where people are suffering the most from HPV-related disease. It is estimated that it will be at least 20 years before the first significant reductions in morbidity and mortality due to preventive vaccinations are observed [9]. Thus, there is a pressing need for novel therapeutic endeavors. So far, over 100 HPV types have been identified, of which HPV16 and HPV18 are the most frequently detected [1]. HPVs are difficult to work with, as they are species-specific and can only replicate in differentiating epithelia [10]. The multiple-subtype complexity of the virus is another difficulty in HPV treatment. Thus, we set our sights on the downstream targets of HPV oncogenes.

In the area of epigenetics, histone modification is important for activating and repressing transcription by altering histone structure. Epigenetic therapy, especially histone modification, has been successful in treating hematopoietic malignancies and many other cancers [11]. However, relevant research in cervical carcinogenesis that avoids the multi-subtype problem of HPV remains inadequate. Lysine-specific demethylase 1 (LSD1), the first histone demethylase discovered, specifically catalyzes the demethylation of mono- or dimethylated histone H3 lysine 4 (H3K4me1, H3K4me2), and histone H3 lysine 9 (H3K9me1, H3K9me2) through a flavin-adenine-dinucleotide-dependent oxidative reaction. LSD1 has been implicated in the maintenance of a variety of cancer types, including neuroblastoma [12], breast cancer [13], prostate cancer [14], colon cancer [15], etc., and elevated LSD1 expression correlates well with tumor progression and unfavorable clinical outcomes [15, 16]. Inhibition or knockdown of LSD1 has been shown to suppress cell growth, migration and invasion in some solid tumors like non-small cell lung cancer [17]. Though LSD1 inhibitors have been clinically researched and applied to treat myelogenous leukemia [18, 19], they have not yet been used to treat solid tumors. In this study, we suggest that LSD1 is a promising therapeutic target because it is downstream of HPV16 E7 and thus avoids the HPV subtype-related restriction.

The invasiveness of epithelial cells depends on the activation of the epithelial–mesenchymal transition (EMT) [20]. The molecular hallmarks of the EMT include downregulation of the epithelial adhesion protein E-cadherin and *de novo* expression of N-cadherin and the mesenchymal intermediate filament proteins Vimentin and fibronectin. Vimentin is one of the most widely expressed and highly conserved proteins of the type III intermediate filament protein family, and is responsible for altering the cellular shape and strengthening the cytoskeleton [21]. Increased Vimentin expression has been reported in various epithelial cancers, and correlates with tumor growth, invasion and poor prognosis [22–24]. Upregulation of Vimentin allows the cellular shape to change and increases cellular motility, strongly signaling the occurrence of the EMT.

Although LSD1 is an important promoter of tumorigenesis, the mechanism resulting in the aberrant expression of LSD1 in tumors remains unclear. In the present study, we validated the high expression of LSD1 in cervical carcinoma, and demonstrated that LSD1 enhances cervical cancer invasion and metastasis. We took an unbiased approach to explore the relationship between LSD1 and HPV16 E7, and discovered that HPV16 E7 influenced both the recruitment of LSD1 to the Vimentin promoter and the demethylation activity of LSD1. All of these data indicated that LSD1 promotes metastasis in cervical cancer cells and is a critical target of HPV16 E7 in the EMT of cervical cancer.

## RESULTS

### Survival analysis based on *LSD1* levels from TCGA data

**Figure 1:**
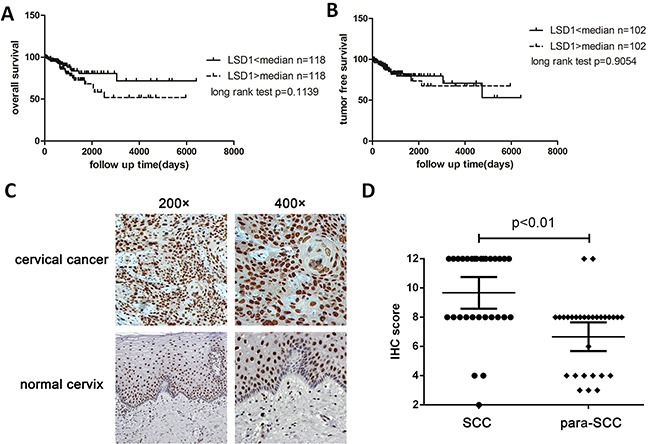
Overall survival and tumor-free survival of LSD1 high expression group and LSD1 low expression **A**. and **B**. Kaplan-Meier plot of the 236 cervical cancer patients according to normalized *LSD1* counts from TCGA RNASeq. The median risk score was used to divide patients into the high- and low-risk groups. Kaplan-Meier curves are shown for A) overall survival and B) tumor-free survival **C**. The expression of LSD1 in cervical tumor tissues and adjacent normal tissues in an immunohistochemical staining assay (×200, ×400). **D**. Scattergram analysis of the expression of LSD1 in 28 pairs of carcinoma tissues and adjacent normal tissues

We downloaded and collected the Level 3 normalized counts of LSD1 and cervical cancer clinical data from The Cancer Genome Atlas (TCGA). For each patient in the cervical cancer cohort (n = 236), a normalized count of RNASeq data was calculated. Normalized counts were dichotomized at the median, and the cohort was divided into two groups – those with relatively low and relatively high expression of *LSD1*. Curves for overall survival and tumor-free survival were plotted according to the Kaplan-Meier method, with *p* values determined by the Log-Rank test. The difference between the two groups was not statistically significant (Figure 1A & 1B). We also acquired the *LSD1* level 3 normalized counts, which represent the *LSD1* RNA levels from 307 cervical cancer specimens from TCGA, and organized them into a heat map according to HPV subtype (Supplementary Figure 1).

### LSD1 protein expression in human cervical tissues

The cellular localization of LSD1 was determined by immunohistochemistry. As expected, the majority of the LSD1 protein was located in the nucleus. We considered both the intensity of the staining and the percentage of positively stained nuclei, and evaluated the expression of LSD1 according to the nuclei present, with the possible scores ranging from 0-9 (Figure 1C). LSD1 was detectable in almost all cervical samples (Supplementary Table 1). Cervical squamous cell carcinoma (SCC) samples exhibited stronger staining of LSD1 (7.9±1.9, Mean±S.D.) than normal cervix (NC) samples (5.6±2.3), cervical intraepithelial neoplasia (CIN) tissues (6.3±2.0) and other cervical carcinoma tissues (5.1±1.6), indicating that LSD1 expression is elevated in SCC (Supplementary Table 2). To determine whether LSD1 protein levels correlated with cervical tumor grades, we performed the following comparisons: NC vs. CIN, NC vs. SCC, and CIN vs. SCC (other cervical carcinoma tissues were omitted). The expression of LSD1 was remarkably stronger in SCC tissues than in NC or CIN tissues (*p*<0.05, Supplementary Table 3), but the difference between the NC and CIN groups was not statistically significant. The positive expression rate of LSD1 in SCC tissues did not differ significantly between patients above or below the age of 45, nor was it significantly related to histopathological grade or TNM classification (*p>*0.05). However, the expression of LSD1 was related to lymph node metastasis (*p*<0.05) (Table 1).

**Table 1:** Relationship between clinical pathologic characteristics and expression of LSD1 in cervical carcinoma tissues

Factors	Number of patients	Negative	Positive			*P*-value
			+	++	+++	
*Age (year)*						
<45	46	0	1	11	34	0.1702
≥45	53	1	3	15	34	
*Histological grade*						
Low (I)	2	1	0	0	1	0.3232
Intermediate (II)	58	0	2	10	46	
High (III)	39	0	2	16	21	
*T stage*						
T1+T2	98	1	4	26	67	0.5419
T3+T4	1	0	0	0	1	
*N stage*						
N0	78	1	2	21	54	0.6458
N1+N2	21	0	2	5	14	
*Lymph node metastasis*						
No	68	1	3	6	58	0.0002
Yes	31	0	1	20	10	

### LSD1 expression in paired cervical carcinoma and adjacent normal tissues

Next, we analyzed the expression of LSD1 in 28 pairs of cervical carcinoma tissues and adjacent normal tissues. The score for LSD1 was significantly higher in SCC tissues than in adjacent normal tissues (7.4±2.1 vs. 5.1±1.7; *p* = 0.0025; Supplementary Table 4, Figure 1C&D). The staining of LSD1 in adjacent normal tissues was heterogeneous and weak compared to that in carcinoma tissues (Figure 1C).

### LSD1 and HPV16 E7 promoted the invasion of cervical cancer cell lines

**Figure 2:**
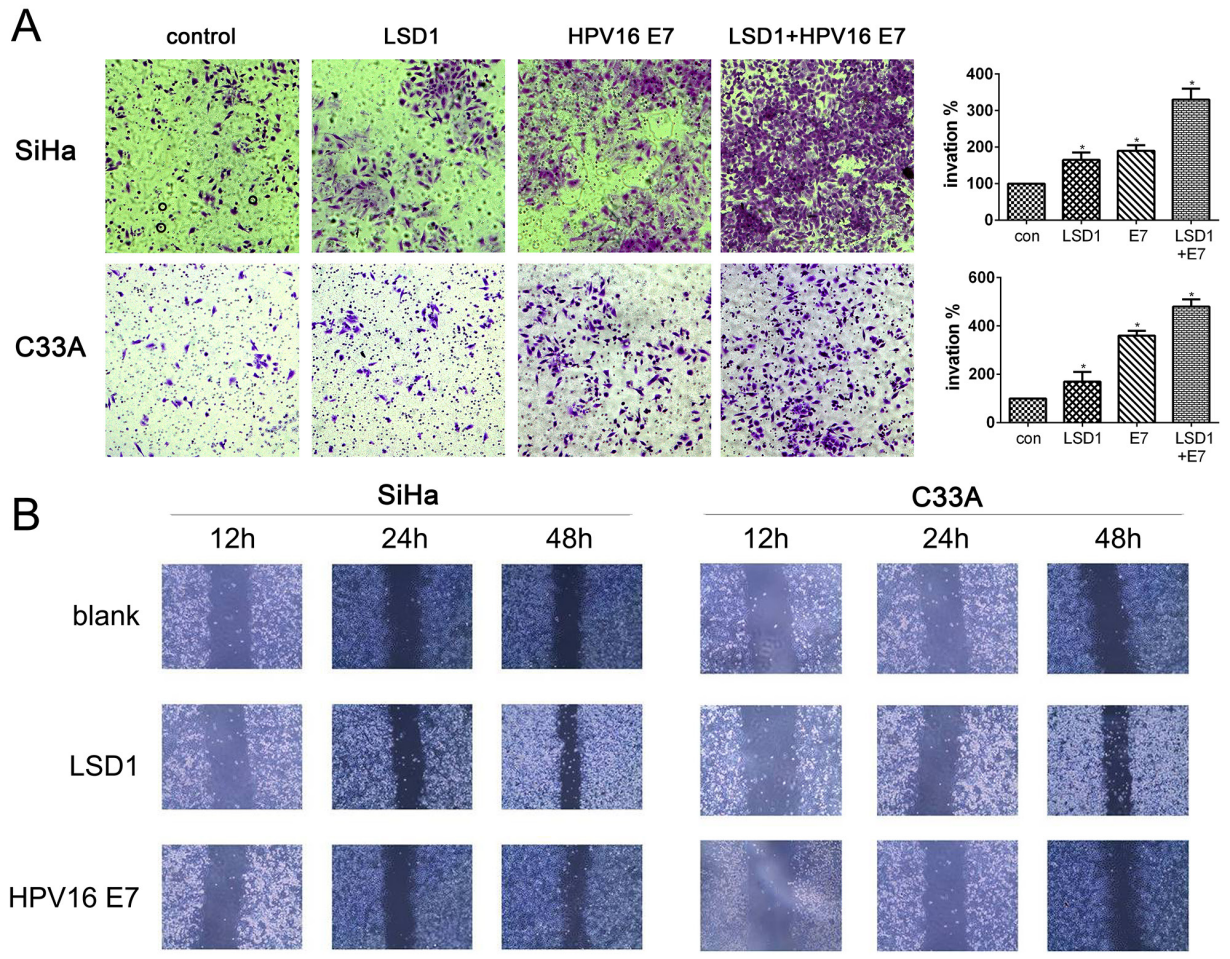
Effect of LSD1 and HPV16 E7 overexpressing on migration and invasion **A**. Effects of LSD1 and HPV16 E7 on the migration of cervical tumor cells in a Transwell assay. Microphotographs display representative fields of lower membranes of the BioCoat chambers; histograms display the number of cells counted by trypan blue exclusion (expressed as a %, with untreated cells as 100%). *p<0.05, compared with the blank group. **B**. Effects of LSD1 and HPV16 E7 on the invasion of cervical tumor cells in a wound-healing assay. Microphotographs display repopulation of the wounded areas of *LSD1*-overexpressing cells, *HPV16 E7*-overexpressing cells, and blank cells.

In order to evaluate the involvement of LSD1 in cervical cancer invasion and metastasis, we first examined the effects of LSD1 and HPV16 E7 on the motility of cervical cancer cells through a cell invasion assay. After *LSD1* and *HPV16 E7* were individually overexpressed in both SiHa and C33A cell lines, the impact of the gain-of-function on the invasive potential of these cells was investigated through Transwell invasion assays. Overexpression of either *LSD1* or *HPV16 E7* increased cell invasion several-fold relative to the level in the blank group (*p*<0.05, Figure 2A). Similarly, 48 hours after the cell monolayer was scratched in a wound-healing assay, *LSD1*-overexpressing and *HPV16 E7*-overexpressing cervical cancer cells filled the wounds at a much faster rate than untreated cells (Figure 2B). Thus, *LSD1*- and *HPV16 E7*-overexpressing cervical cancer cells exhibited remarkably stronger invasion and migration capabilities than cells in the blank group *in vitro*.

### LSD1 and HPV16 E7 reduced E-cadherin expression and increased Vimentin expression

**Figure 3:**
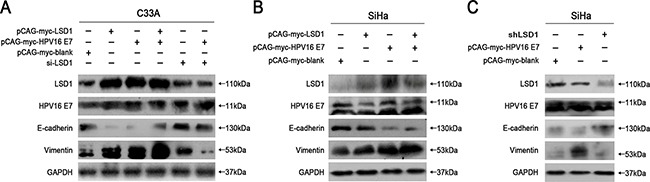
Effects of LSD1 and HPV16 E7 gene modulation on the EMT-related genes expressions The expression of the hallmarks of the EMT (E-cadherin, Vimentin) when SiHa and C33A cells were transfected with an *LSD1* overexpression plasmid, *HPV16 E7* overexpression plasmid, *LSD1* siRNA or *LSD1* shRNA. Transfection of SiHa and C33A cells with the *LSD1* overexpression plasmid upregulated Vimentin and downregulated E-cadherin, while the knockdown of LSD1 had the opposite effects. Stronger expression of LSD1 was detected in *HPV16 E7*-overexpressing cells compared to the blank group.

Corresponding to the invasion assay, we found that overexpression of *LSD1* and *HPV16 E7*, respectively and cooperatively, increased the expression of the mesenchymal marker Vimentin and reduced the expression of the epithelial marker E-cadherin in C33A and SiHa cells (Figure 3A & 3B). Notably, stronger expression of LSD1 was detected in *HPV16 E7*-overexpressing cells compared to the blank group, suggesting that HPV16 E7 enhances the expression of LSD1 (Figure 3A & 3B).

To further characterize the function of LSD1 in the HPV16 E7-induced EMT, we knocked down LSD1 in cervical cancer cells. Knockdown of LSD1 hindered the EMT, based on the observation that it reduced Vimentin expression and increased E-cadherin expression relative to their expression in the blank group (Figure 3A & 3C). Knockdown of LSD1 in *HPV16 E7*-overexpressing cervical cancer cells also reduced the expression of Vimentin and increased the expression of E-cadherin compared to their expression in the *HPV16 E7*-overexpressing control group, resulting in levels similar to those in the blank group (Figure 3A), indicating that the enhancement of the EMT by HPV16 E7 was diminished by reduced LSD1 expression. These results suggested a positive and critical function of LSD1 in the HPV16 E7-induced EMT in cervical cancer cells.

### LSD1 co-immunoprecipitated with HPV16 E7

**Figure 4:**
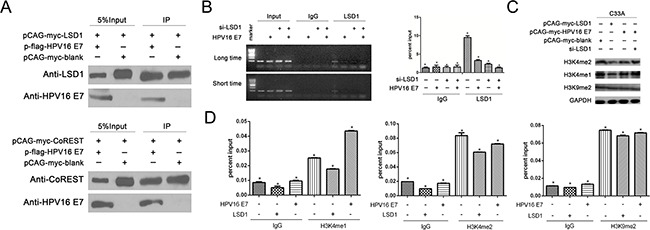
The effect of LSD1 and HPV16 E7 gene modulation on epigenetic change on the Viemntin promoter **A**. Association of LSD1 with HPV16 E7 in C33A cells. Immunoprecipitation with an antibody against LSD1, followed by immunoblotting with an antibody against HPV16 E7, demonstrated that HPV16 E7 co-immunoprecipitated with LSD1. Association of Co-Rest with HPV16 E7 in C33A cells. Immunoprecipitation with an antibody against Co-REST, followed by immunoblotting with an antibody against HPV16 E7, demonstrated that HPV16 E7 co-immunoprecipitated with Co-REST. **B**. HPV16 E7 suppressed the recruitment of LSD1 to the Vimentin promoter. Occupancy of LSD1 at the Vimentin promoter was markedly lower in *HPV16 E7*-overexpressing cells and siLSD1 C33A cells than in blank cells, as shown by ChIP analysis with an LSD1 antibody. **C**. The histone change caused by LSD1 and its relationship with HPV16 E7. In Western blotting, H3K4me1 and H3K4me2 protein levels were reduced in *LSD1*-overexpressing C33A cells, but not in *HPV16 E7*-overexpressing cells. Knockdown of LSD1 induced the expression of H3K4me1 and H3K4me2. H3K9me2 protein levels remained the same in all groups. **D**. In ChIP assays, the levels of H3K4me1 and H3K4me2 at the Vimentin promoter were lower in *LSD1*-overexpressing cells than in blank cells. The level of H3K4me1 at the Vimentin promoter was greater in *HPV16 E7*-overexpressing cells than in blank cells and *LSD1*-overexpressing cells. The level of H3K4me2 at the Vimentin promoter was greater in *HPV16 E7*-overexpressing cells than in *LSD1*-overexpressing cells. The level of H3K9me2 remained the same.

As we discovered that HPV16 E7 enhanced LSD1 expression in the above experiments, we performed co-immunoprecipitation assays to examine whether HPV16 E7 and LSD1 might form a complex. Immunoprecipitation with antibodies against LSD1, followed by immunoblotting with antibodies against HPV16 E7, demonstrated that HPV16 E7 co-immunoprecipitated with LSD1 in C33A cells. HPV16 E7 was detected in the immunoprecipitated fraction obtained from cells expressing pCAG-myc-LSD1 (Figure 4A). In addition, we immunoprecipitated Co-REST, and found that HPV16 E7 also co-immunoprecipitated with this protein in C33A cells (Figure 4A). This evidence suggested that HPV16 E7 forms a protein complex with both LSD1 and Co-REST.

### HPV16 E7 suppressed the recruitment of LSD1 to the Vimentin promoter

LSD1 is a component of multiple transcription factor complexes, and thus has the ability to repress or activate gene transcription. Since we found that gain or loss of LSD1 respectively elevated or reduced the expression of Vimentin in cervical cancer cells (Figure 4A–4C), we next performed chromatin immunoprecipitation (ChIP) to investigate whether LSD1 could bind to the promoter of the mesenchymal gene Vimentin to modify histones and thus enhance gene expression. Chromatin from C33A cells was precipitated with control immunoglobulin G (IgG) and an anti-LSD1 antibody, and RT-PCR was performed on the recovered DNA so that the enrichment of the proximal promoter regions of Vimentin could be determined, relative to three other gene sequences within 800 bp upstream of the target gene. Occupancy of LSD1 was detected specifically at the transcription start site of Vimentin in cervical cancer cells (Supplementary Figure 4). These data suggested that LSD1 binds to the promoter of Vimentin.

Given our observations that HPV16 E7 was able to form a complex with LSD1, and that LSD1 could bind to the promoter of Vimentin, it seemed plausible that the formation of a complex of HPV16 E7 and LSD1 at the Vimentin promoter could influence the transcription of Vimentin. Thus, we further investigated the impact of HPV16 E7 on the Vimentin promoter and the recruitment of LSD1 to it. We performed a ChIP assay to compare the enrichment of LSD1 at the Vimentin promoter in four groups of C33A cells: blank, *HPV16 E7*-overexpressing, LSD1-knockdown, and both LSD1-knockdown and *HPV16 E7*-overexpressing. Using an antibody specific for LSD1, we detected significantly lower enrichment of LSD1 at the promoter of the Vimentin gene in *HPV16 E7*-overexpressing cells than in blank cells (Figure 4B). As predicted, LSD1 enrichment at the Vimentin promoter was significantly reduced in the LSD1-knockdown group and the both LSD1-knockdown and *HPV16 E7*-overexpressing group (Figure 4B). These results suggested that HPV16 E7 suppresses the enrichment of LSD1 at the Vimentin promoter.

### The histone modification caused by LSD1 and its relationship with HPV16 E7

LSD1 specifically catalyzes the demethylation of mono- or dimethylated histone H3 lysine 4 (H3K4me1, H3K4me2) and histone H3 lysine 9 (H3K9) through a redox process [25]. Considering this well-established mechanism, and the direct protein-protein interaction between LSD1 and HPV16 E7 (Figure 4A), we examined the histone modification induced by LSD1 and the effect of HPV16 E7 on it. In Western Blotting, reduced levels of H3K4me1 and H3K4me2 were only detected in *LSD1*-overexpressing cervical cells, not in *HPV16 E7*-overexpressing cells (Figure 4C). Additionally, knockdown of LSD1 had the opposite effect (Figure 4C). These results suggested that LSD1 indeed demethylated H3K4me1 and H3K4me2 in the *LSD1*-overexpressing cervical cells. However, there was not much change in the expression of H3K4me1 and H3K4me2 in *HPV16 E7*-overexpressing cells relative to their expression in the blank group (Figure 4C). Thus, we speculated that although HPV16 E7 boosts LSD1 expression, it might not enhance its demethylation of H3K4me1 and H3K4me2. On the other hand, neither *LSD1* nor *HPV16 E7* overexpression affected the level of H3K9me2 in cervical cancer cells (Figure 4C).

Given that Western Blotting assays can only reflect the overall protein level of a histone marker, it was necessary to conduct a ChIP assay to analyze the particular histone demethylation at the Vimentin promoter and explore the influence of LSD and HPV16 E7 on Vimentin gene transcription. Thus, we performed a ChIP assay examining H3K4 methylation marks (H3K4me1, H3K4me2) and H3K9 methylation marks (H3K9me2), targets of demethylation by LSD1, at the Vimentin promoter. Using an antibody specific for H3K4me1 and H3K4me2, we detected lower levels of H3K4me1 and H3K4me2 at the Vimentin promoter in *LSD1*-overexpressing C33A cells than in cells from the blank group (Figure 4D, Supplementary Figure 5), confirming that LSD1 demethylated histone H3K4me1 and H3K4me2 at the Vimentin promoter. Moreover, the levels of H3K4me1 and H3K4me2 were greater in *HPV16 E7*-overexpressing cells than in *LSD1*-overexpressing cells (Figure 4D, Supplementary Figure 5), suggesting that HPV16 E7 did not enhance the ability of LSD1 to demethylate H3K4me1 and H3K4me2 at the Vimentin promoter, but rather rescued the methylation of these two histone sites. These results were consistent with our speculation from the Western Blotting assay (Figure 4C) that although HPV16 E7 boosted LSD1 expression, it did not enhance the LSD1-induced demethylation of H3K4me1 and H3K4me2. As HPV16 E7 did not facilitate the enrichment of LSD1 at the Vimentin promoter (Figure 4B) or stimulate its enzymatic activity against H3K4me1 and H3K4me2 at the Vimentin promoter (Figure 4D), we concluded that HPV16 E7 suppressed the demethylation function of LSD1 and hindered its enrichment at the Vimentin promoter. Additionally, we found no significant change in H3K9me2 levels at the Vimentin promoter in any group (Figure 4D, Supplementary Figure 5).

### LSD1 promotes the metastasis of cervical cancer cells *in vivo*

**Figure 5:**
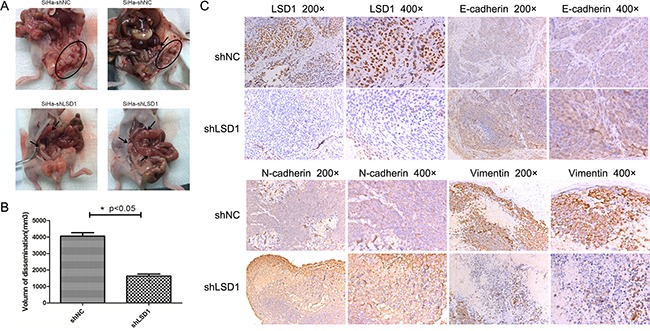
Effects of LSD1 knockdown on peritoneal disseminated tumor growth **A**. Laparotomy of nude mice to explore disseminated tumors >1 mm in diameter. Arrows and the circled area display solitary tumors and the tumor cluster, respectively. **B**. Volume of disseminated tumors in shNC and shLSD1 groups. **C**. Immunohistochemical staining of LSD1, Vimentin, N-cadherin and E-cadherin in shNC and shLSD1 disseminated tumors. *p<0.05

To test whether LSD1 potentiates SiHa cell metastasis *in vivo*, we performed a peritoneal dissemination assay. SiHa cells were stably transfected with short hairpin LSD1 (shLSD1) or shNC and intraperitoneally injected into nude mice. Pathological anatomical analysis of the disseminated tumors was carried out five weeks after injection, and significantly lower tumor growth was observed in the shLSD1 group than in the shNC group (Figure 5A). Furthermore, peritoneal metastasis in the shNC group was mostly located near the uterus. LSD1 expression was verified to be depleted in the shLSD1 disseminated tumors, and weaker staining of Vimentin and stronger staining of E-cadherin and N-cadherin were detected in shLSD1 tumors than in shNC tumors (Figure 5B), demonstrating that LSD1 facilitates metastasis *in vivo*.

## DISCUSSION

LSD1 contains three domains: an N-terminal SWIRM structural domain, a tower domain which provides a surface for interaction with other proteins (e.g., Co-REST and MTA2), and an amine oxidase domain which harbors the demethylase activity [26, 27]. Binding of Co-REST to the tower domain of LSD1 reduces the proteasomal degradation of LSD1, helps to hitch the LSD1 complex to chromatin, and activate the histone demethylase activity of LSD1 [28]. Recently, high levels of LSD1 protein have been found in several types of solid tumors and have been associated with poor prognosis; for instance, LSD1 expression gradually increases during tumor progression from pre-invasive ductal carcinoma in situ to invasive ductal breast carcinoma [16]. In addition, overexpression of LSD1 protein correlates with disease progression and poor prognosis in hepatocellular carcinoma patients [29], and promotes recurrence and elevated VEGF-A expression in prostate cancer patients [30].

LSD1 overexpression has been found to contribute to human carcinogenesis through chromatin modification, while its inhibition reduces or blocks cell growth in many tumors31. However, an exception was reported – namely, that LSD1, as an integral component of the Mi-2/nucleosome remodeling and deacetylase complex, inhibited the invasion of breast cancer cells *in vitro* and suppressed breast cancer metastasis *in vivo* [32]. Our research showed for the first time that the protein expression of LSD1 was stronger in cervical SCC tissues than in CIN tissues, normal cervical tissues, or adjacent normal tissues to the SCC tissues. Additionally, LSD1 expression correlated with lymph node metastasis, though LSD1 RNA expression did not significantly correlate with overall survival or tumor-free survival from TCGA data. LSD1 enhanced cervical cancer cell invasion and metastasis both *in vitro* and *in vivo*; however, N-cadherin expression increased in shLSD1 SiHa cell disseminated tumors. N-cadherin is mainly expressed in neural and mesenchymal tissues, and this mesenchymal marker could be involved in inducing the EMT in pancreatic cancer or other carcinomas [33]. However, low levels of positive N-cadherin expression in CIN and SCC tissues have been reported and considered not to be related to CIN or cervical cancer [34]. Thus, it is conceivable that the expression of N-cadherin did not decrease when the EMT in cervical cancer was hindered by the knockdown of LSD1.

As LSD1 is known to function as a tumor promoter, previous studies have examined whether post-translational modification by LSD1 specifically induces the EMT. Lin *et al*. reported that LSD1 was physically recruited to the E-cadherin promoter with the cooperation of the transcription factor Snai1, where it then removed two methyl groups from lysine 4 on histone H3 (H3K4me2) and repressed E-cadherin transcription, ultimately enhancing the EMT [35, 36]. In the current study, we observed that both HPV16 E7 and LSD1 induced metastasis and invasion, downregulated E-cadherin and upregulated Vimentin *in vitro*. Moreover, knocking down LSD1 in cervical tumor cells restored the expression of E-cadherin and reduced the expression of Vimentin. Since Vimentin upregulation is associated with poor prognosis and lower survival in prostate and colorectal cancer types [22], it is clinically relevant that LSD1 upregulates Vimentin during the EMT in HPV16 E7-induced cervical cancer. This was not the first evidence that Vimentin undergoes epigenetic modifications. Not only did Jin *et al*. identify Vimentin as a target gene of LSD1 [37], but Wu *et al*. also demonstrated that the transcription factor ZBP-89 recruits histone deacetylase 1 to the Vimentin promoter, thus reducing Vimentin expression [38]. Our data built upon these studies, as we further elucidated that LSD1 was recruited to Vimentin promoter, demethylated H3K4me1 and H3K4me2, activated Vimentin transcription and thus served as a critical positive regulator of the EMT in cervical cancer.

**Figure 6:**
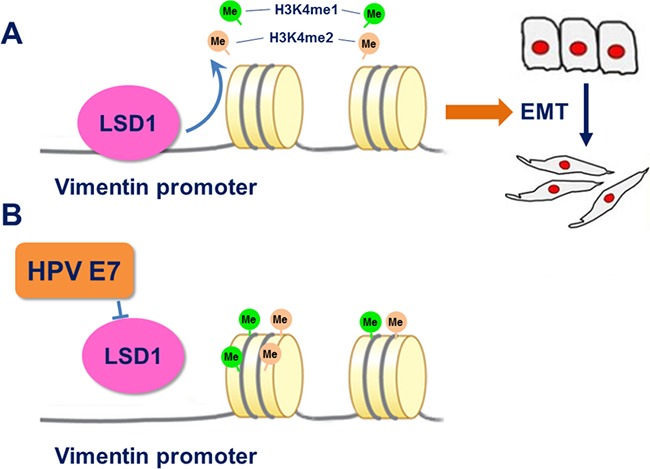
**A**. After binding to the Vimentin promoter, LSD1 demethylated H3K4me1 and H3K4me2, activated the transcription of Vimentin, and induced the EMT. **B**. HPV16 E7 suppressed the enrichment of LSD1 on the Vimentin promoter and rescued the methylation of H3K4me1 and H3K4me2.

It is well established that stable expression of the oncogenes *HPV16 E6* and *E7* induces the EMT in cervical cancer cells through a series of mechanisms that ultimately increase the expression of the EMT-activating transcriptional factors Slug, Twist, ZEB1, ZEB2, α-SMA, Vimentin and fibronectin, and reduce the protein expression of E-cadherin through a DNA methyltransferase 1-dependent mechanism [39–42]. Malignancy further progresses as other genetic and epigenetic alterations occur, such as the interplay among the HPV oncogenes, DNA methyltransferase and histone modification enzymes [43–46]. E7 facilitates the removal of histone deacetylase from certain promoters, such as that of *E2F2*, to increase transcriptional activity [47]. E7 can also displace histone deacetylases from Rb protein and subsequently promote H3 acetylation in human foreskin keratinocytes [48]. All of these interplays above have implications for the role of the epigenetic regulation played by HPV viral genome [49]. In our study, we demonstrated that the introduction of HPV16 E7 could enhance the expression of LSD1, and that these two proteins had the same effect on the expression of EMT markers and formed a complex. Notably, HPV16 E7 attenuated the recruitment of LSD1 to the Vimentin promoter and also suppressed the demethylation of H3K4me1 and H3K4me2 (Figure 6). These data further illustrated that LSD1-dependent histone demethylation at Vimentin promoter served as the downstream target of HPV16 E7, and more importantly facilitated the EMT in cervical cancer. HPV16 E7 promoted cell invasion and migration by promoting the expression of and binding with the EMT-inducing transcription factor LSD1.

LSD1 can have different effects on transcription by demethylating different histones and associating with different complexes [50]. As a component of co-repressor complexes including CoREST, CtBP, and a subset of histone deacetylases [50], LSD1 represses transcription by demethylating H3K4me1 and H3K4me2 [26, 51]. However, when co-localized with the androgen receptor, LSD1 functions broadly as a transcriptional activator by removing repressive methyl histone marks from H3K9, ultimately de-repressing androgen receptor target genes [52]. LSD1 also co-activates AR-induced transcription by binding to other proteins like FOXA1 [53]. Therefore, depending on its interacting partners and the target histone, LSD1 could either silence or activate gene expression. Furthermore, the same demethylation event may have different functional outcomes at different locations along the gene. For example, the repressive H3K9 methylation, when found within the body of the gene, has been shown to positively regulate transcription [54]. Thus, LSD1 has dual functions in its regulation of the transcription of some genes.

Jin *et al*. identified the genes and gene families of which expression was directly or indirectly affected by LSD1 in a microarray analysis of colorectal cancer [37]. Among these genes were Vimentin, vesicle amine transport protein 1 homologue-like, interferon α-inducible protein 6, and interleukin 8. When the authors mapped the LSD1 localization at several sites in the promoters of these genes, they observed higher LSD1 occupancy at sites near the transcription start site than at more distal 5’ regions (approximately 3000 bp upstream of the transcription start site). They also demonstrated that Vimentin RNA expression was higher in LSD1 knockdown cells than in wildtype cells in colorectal cancer. In contrast, we found that overexpression of *LSD1* increased Vimentin expression, while knockdown of LSD1 attenuated Vimentin expression at the protein level in cervical cancer. This also attests to the complicated and unpredictable effects of LSD1 on the transcription of different target genes in different cancer types.

Our study revealed that LSD1 bound to the Vimentin promoter and demethylated the histone marks H3K4me1 and H3K4me2. We speculated that this is one of the mechanisms whereby LSD1 promotes Vimentin expression in cervical cancer. Vimentin may be modified directly or indirectly by other transcription factors by different mechanisms in cervical cancer cells. These results underscore the fact that methylation-dependent transcriptional modification in cancer cells is not completely understood. The site and the extent of methylation, as well as the precise place where methylation occurs at the gene locus, can all impact the functional outcome. The important finding of this study is that Vimentin is one of the genes through which LSD1 promotes the EMT in cervical cancer.

Although LSD1 is known to regulate the expression of several downstream targets, the upstream regulators of LSD1 have not been extensively explored. We discovered that HPV16 E7 is an upstream factor of LSD1 that promotes LSD1 expression and nevertheless restrains histone methylation by suppressing the recruitment of LSD1 at the Vimentin promoter. Likewise, knockdown of LSD1 attenuated the HPV16 E7-dependent EMT. Thus, LSD1 could be a promising target in treatments for HPV-induced cervical cancer metastasis designed to avoid HPV restrictions.

In conclusion, we proposed a novel mechanism whereby ectopic expression of LSD1 promoted the EMT in cervical cancer, and discovered intriguing functions of HPV16 E7 and LSD1 in the promotion of Vimentin transcription in cervical cancer. We demonstrated that LSD1 binds to its upstream regulator (HPV16 E7), and its downstream target (Vimentin), to promote the EMT in cervical cancer. LSD1 thus appears to be an attractive therapeutic target for reversing the HPV16 E7-induced EMT and tumor metastasis. Further functional analyses of the LSD1 protein, especially the histone modification influence on different target genes in the individual human cervical carcinogenesis, may assist in the development of novel therapeutic strategies, and warrant further investigation.

## MATERIALS AND METHODS

### Ethics

This study was conducted according to the tenets of the Declaration of Helsinki for the use of human subjects, and all the specimens for immunohistochemical staining were approved by the Ethics Committee of Shanghai First People's Hospital, Shanghai Jiaotong University, Shanghai, China (Permit Number: 2012K038). Informed consent was obtained from all patients.

### TCGA data

Level 3 normalized counts of LSD1 (RNA-Seq; Illumina) and cervical cancer clinical data were downloaded from TCGA and analyzed in the R statistical environment. Survival rates were calculated by the Kaplan–Meier method, and the log-rank test was used to compare the survival curves. The heat map of the LSD1 normalized counts from level 3 RNASeq TCGA data was made in Excel; the green color is aligned with the largest normalized count and the deepest red color is aligned with the smallest normalized count.

### Tissue samples

Archived cervical specimens representing a wide range of cervical disease processes, including 45 normal cervical tissues, 35 CIN tissues, 99 cervical SCC tissues, and 14 cervical cancer tissues of other histological types, were selected for analysis from the case files of the Department of Obstetrics and Gynecology at Shanghai Jiao Tong University, Affiliated Shanghai General Hospital between February 2009 and August 2012. Twenty-eight pairs of cervical SCC tissues and adjacent normal tissues were also included. Pathological diagnoses of cervical samples were made in a double-blinded manner by two experienced gynecologic pathologists (J.T. Xu and Z.L. Chen) according to the World Health Organization classification.

### Immunohistochemical staining

Immunohistochemical analysis of LSD1 protein was performed as previously described [55]. The sections were incubated with a rabbit anti-human LSD1 antibody (diluted 1:100; Sigma, St. Louis, MO, USA). Expression of LSD1 protein was assessed by a semi-quantitative method: the sections were assessed for the intensity of staining (0-3) and the percentage of positively stained nuclei (0-3). The index of LSD1 expression was calculated as the percentage × intensity of the staining. Therefore, a score 0 was considered negative (−), scores of 1-3 were considered weakly positive (+), 4-6 were positive (++), and 7-9 were strongly positive (+++).

### Cell lines and cell culture

Human cervical carcinoma lines - HPV 16(+) SiHa and HPV(−) C33A (Supplementary Figure 2) -were purchased from ATCC and maintained in Dulbecco's modified Eagle's medium (DMEM)/F-12 1:1 medium (GIBCO from Thermo Fisher,USA) with 10% fetal bovine serum (FBS; GIBCO), 100 U/mL penicillin, sodium pyruvate and L-glutamine in a humidified atmosphere of 5% CO_2_ at 37°C. The cell lines were frozen in liquid nitrogen in Dr. Wu's lab for eight months and no authentication was performed.

### Overexpression plasmids, siRNA and lentiviral shRNA transfection

The overexpression plasmids (pCAG-myc-LSD1, pCAG-myc-HPV16E7, and pCAG-myc-blank) were maintained in Dr. Wu's lab, and the *LSD1* small interfering RNA (siRNA) was purchased from Shanghai GenePharma Co. Ltd. We used siRNA to knock down the *LSD1* gene in the C33A cell line (Supplementary Figure 3). The sequences were as follows: siLSD1-1, CCACGAGUCAAACCUUUAUTT, AUAAAGGUUUGACUCGUGGTT; siLSD1-2: GCCACCCAGAGAUAUUACUTT, AGUAAUAUCU CUGGGUGGCTT. The acute siRNA and stable shRNA plasmid transfection experiments were performed as previously described [56]. *LSD1* shRNA constructs were obtained from Professor Xiaobing Shi at the Center for Epigenetics in the UT M.D. Anderson Cancer Center. We tried three different *LSD1* shRNA duplexes; however, only pLKO-puro-927 worked well, such that LSD1 was knocked down in seven days upon the addition of Doxycycline (Supplementary Figure 3). The pLKO-puro-927 sequences were: CCGGGCACCTTATAACAGTGATACTCTCGAGAGTATCACTGTTATAAGGTGCTTTTT. SiHa cells were transduced with 5*10^5^ transducing units/mL of lentiviral particles. Antibiotic selection (2 μg/mL puromycin) was initiated for seven days, 24 hours after transduction. As a result, a stable SiHa shLSD1 cell line transduced with pLKO-puro-927 was generated. Cells transduced with pLKO-puro-ctrl-shRNA (SiHa shNC) were used as controls. Doxycycline (1 μg/mL) was added to the cell culture medium to start the Tet-on system and thus knock down LSD1.

### Western blotting analysis

Western blotting was performed as previously described [57]. Briefly, 60 μg protein was separated by SDS-PAGE and transferred to a polyvinylidene fluoride membrane. Membranes were blocked with 5% skim milk for 2 hours and incubated for 15 hours with the following rabbit monoclonal primary antibodies: anti-LSD1 (diluted 1:500; Sigma, St. Louis, MO, USA), anti-HPV16 E7 (diluted 1:100; Bioss, Shanghai, China), anti-Vimentin (diluted 1:500; Cell Signaling Technology, Beverley, MA, USA), anti-E-cadherin (diluted 1:500; Cell Signaling Technology), anti-GAPDH (Epitomics), anti-H3K4me1 (Abcam, Cambridge, UK), anti-H3K4me2 (Abcam) and anti-H3K9me2 (Abcam), followed by 1 hour of incubation with the appropriate secondary antibody.

### Migration and invasion assays

For Transwell assays, cells (100 cells/chamber) were seeded on top of BioCoat Matrigel invasion chambers (BD Biosciences). The medium was supplemented with 2% heat-inactivated FBS in the upper chamber, and with 20% FBS in the lower chamber as a chemo-attractant. Cells that invaded through the Matrigel-coated membrane after 36 hours were fixed with paraformaldehyde for 15 minutes, stained with crystal violet for 20 minutes, and washed eight times with PBS. The upper surface of the membrane was scrubbed with a cotton-tipped swab to remove the non-invading cells. Five fields for each chamber were photographed with a digital camera mounted on an inverted microscope (magnification×100) and the number of invading cells in each field was measured.

For the wound-healing assay, cells were plated to confluence in a six-well plate, and the surface was scratched with a pipette tip. Forty-eight hours later, we photographed the plates with a digital camera mounted on an inverted microscope (magnification×50) to evaluate the migration rate. Two independent experiments were carried out.

### Co-immunoprecipitation and ChIP

Co-immunoprecipitation assays were performed as previously described [58]. For the ChIP assay, briefly, cells were cross-linked with 1% formaldehyde for 15 min. Cross-linked cells were collected after being washed in PBS. Cell pellets were washed in washing buffer (0.5% TritonX-100, 15 mM EDTA, 1 mM EGTA, 15 mM Tris pH 7.8), resuspended in sonication buffer (1 mM EDTA, 1 mM EGTA, 15 mM Tris pH 7.8), mixed, and sonicated. The sonicated samples were diluted in ChIP buffer (0.05% SDS, 1.5% TritonX-100, 2.0 mM EDTA, 20 mM Tris pH 7.8, 100 mM NaCl) and incubated with a specific antibody. The immunoprecipitates were serially washed to remove non-specific binding. After reverse-crosslinking, the DNA samples were purified and analyzed by quantitative real-time PCR. The primer sequences for the Vimentin promoter were GCTGTAAGTTGGTAGCACTGA and TTCTGTCGAGGGACCTAACGF. The three gene sequences tested as controls were within 800 bp upstream of the target gene in the open reading frame; we picked one sequence every 250-300 bp. The final results represent the percentage of input chromatin, and error bars indicate the standard deviations (S.D.) from triplicate experiments.

### Peritoneal disseminated tumor growth assay

Female athymic nude mice 3-4 weeks of age were purchased from the Shanghai Experimental Animal Center of the Chinese Academy of Science. For the peritoneal disseminated tumor growth assay, mice were randomly divided into two groups (five in each group). SiHa cells transfected with shNC or shLSD1 were injected intraperitoneally into each group at 2 × 10^6^ cells per mouse. In five weeks after injection, the mice were euthanized and laparotomy was performed to detect any disseminated tumors >1 mm in diameter. The volume of dissemination was calculated as the sum of all disseminated tumors. The experiment was carried out in strict accordance with the Guide for the Care and Use of Laboratory Animals and was approved by the Department of Laboratory Animal Science at Shanghai Jiao Tong University School of Medicine.

### Statistical analysis

The statistical significance of the difference of LSD1 expression in the immunohistochemically stained cervical tissues was calculated by one-way analysis of variance. Student's t-test was used to analyze the comparison of LSD1 scores in SCC tissues and adjacent normal tissues, the results of the Western blotting, cell invasion, *in vivo* and ChIP experiments. A two-sided test with *p* < 0.05 was considered statistically significant. All statistical analyses were performed with SAS Release 8.02 (SAS Institute Inc., Cary, NC, USA).

## SUPPLEMENTARY MATERIALS FIGURES AND TABLES


