# Supplementary material for: LSD1 binds to HPV16 E7 and promotes the epithelial-mesenchymal transition in cervical cancer by demethylating histones at the Vimentin promoter
Source: Oncotarget. 2016 Nov 23;8(7):11329–42. doi: 10.18632/oncotarget.13516 (PMC5355268; doi:10.18632/oncotarget.13516)
Supplement: Supplementary file 1 [file oncotarget-08-11329-s001.pdf]

LSD1 binds to HPV16 E7 and promotes the epithelial-mesenchymal transition in cervical cancer by demethylating histones at the Vimentin promoter

SUPPLEMENTARY FIGURES AND TABLES

HPV16	2164.745	1817.204	1346.818	1821.584	3455.653	1948.341	3185.587	1943.422	1427.785	1668.758
	2562.395	2886.296	1263.036	6033.528	1738.794	2097.893	2367.288	1633.006	7091.513	3170.461
	2659.852	2612.212	2928.888	2016.297	3246.571	3158.339	2253.429	3781.818	2492.558	757.9099
	5860.942	1987.83	1935.721	1231.681	4624.11	2661.454	2702.186	4232.775	2884.654	2808.372
	1531.816	1735.235	2241.265	2607.668	1873.549	1595.191	2200.583	3255.566	2815.556	2682.124
	2060.691	2534.939	1805.245	3029.792	3219.085	1733.399	2045.234	1903.352	2272.896	1889.854
	2020.298	1971.02	1709.043	1958.219	2336.172	2732.145	2231	1643.461	1793.159	2347.768
	1481.904	2543.045	2107.908	3357.487	2548.497	3092.984	3314.246	2576.242	2543.993	3630.823
	3042.308	3664.819	2236.797	2665.881	1617.422	2954.254	2078.947	2724.188	2202.363	3342.719
	2429.562	2000.775	1878.478	2852.071	1990.077	1691.522	2402.416	2542.26	2374.459	1477.408
	2696.594	3186.4	2310.355	1751.129	2284.058	1620.557	2242.32	2729.069	2458.846	2116.969
	2954.869	3106.656	1295.469	2542.281	2092.026	2042.113	2442.078	2215.356	3591.653	3132.547
	2343.159	3884.037	2871.582	2843.181	2063.971	2892.823	2105.551	2510.417	2202.419	1656.576
	2316.492	3638.642	2707.755	2342.925	1888.582	2161.253	2030.62	2619.948	2566.976	2225.139
	1166.728	2164.912	2434.474	5222.222	1985.586	1436.245	3797.686	2431.133	2979.179	2265.557
	1712.398	1502.576	3261.918	2655.5	2279.73	3340.497	1646.382	1429.36	1858.124	1774.41
	1647.704	4916.522	1849.932	2967.31	1728.192	1897.477	5154.964	2144.867	1577.087	3125.238
	2439.671	2104.491	1893.723	1874.665						
HPV18	3154.542	3641.808	1515.848	2310.124	1093.113	1469.67	1713.048	1855.756	4099.734	1386.055
	3559.794	2703.672	1818.182	1609.218	2865.984	2545.523	2373.222	3004.519	1684.753	1888.929
	2394.459	2725.331	1991.206	2197.088	3406.733	4591.331	3361.065	1975.728	2260.364	2260.364
	657.1647	3002.439	2701.686	2250.497	1934.496	2568.334	2921.722	1880.307	1956.511	2252.623
HPV45	930.5267	2370.254	2709.707	2534.122	2015.069	1730.238	2297.634	3022.334	1270.237	2815.337
	2938.45	2020.809	2767.104	5597.865	3851.184	2357.424	1355.54	2553.204	1707.488	4247.896
	1703.077	2556.183								
HPV33	2432.13	2091.109	3120.362	2311.529	2473.665	2158.14	2324.836	2119.983	1408.008	
HPV58	1128.311	2858.371	2366.829	2376.098	2291.37	2014.21	1618.629			
HPV52	1697.445	2044.625	2234.076	2139.675	3684.375	2006.006	4102.702			
HPV35	2949.153	2790.045	3892.977	1958.556	2585.46	2104.798				
HPV31	2625.671	1950.943	1299.63	2647.585	2553.647					
HPV39	1697.842	2086.379	4097.3	1577.537	2608.053					
HPV59	1868.574	2101.489	1554.842							
HPV73	2580.611	1206.448								
HPV68	1188.425	1081.295								
HPV51	3043.692									
HPV56	4839.733									
HPV-	2264.351	3321.83	3094.797	1934.15	4000.556	2589.056	2023.237	4510.076	3353.836	1828.154
	4086.348	2522.704	2999.424	3108.258	4871.563	2112.782	3276.191	4409.535	2053.58	3526.625
	4274.258	2592.195	2763.131							

Supplementary Figure 1: The heatmap made with LSD1 normalized counts from 307 cervical cancer specimens in TCGA data distributed according to different HPV infection situation. The green color is aligned to the biggest normalized count and the deepest red is aligned to the smallest normalized count.

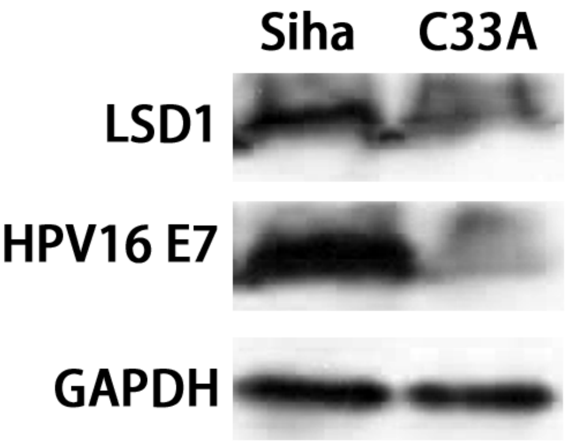

Supplementary Figure 2: The expression of LSD1 and HPV16 E7 in SiHa and C33A cells line by Western Blot.

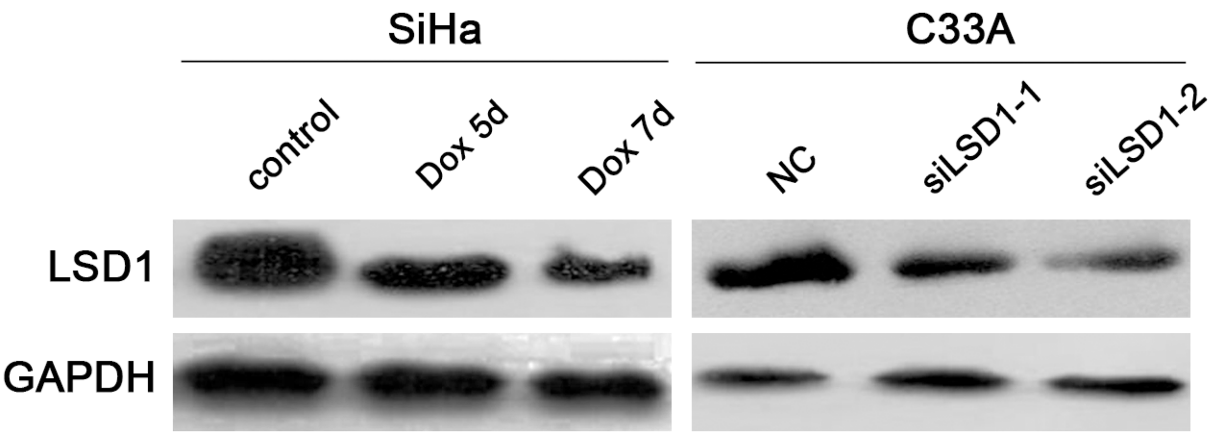

Supplementary Figure 3: Western Blot showed the knock-down effect of LSD1 in SiHa and C33A cells line with virus vector and siRNA respectively.

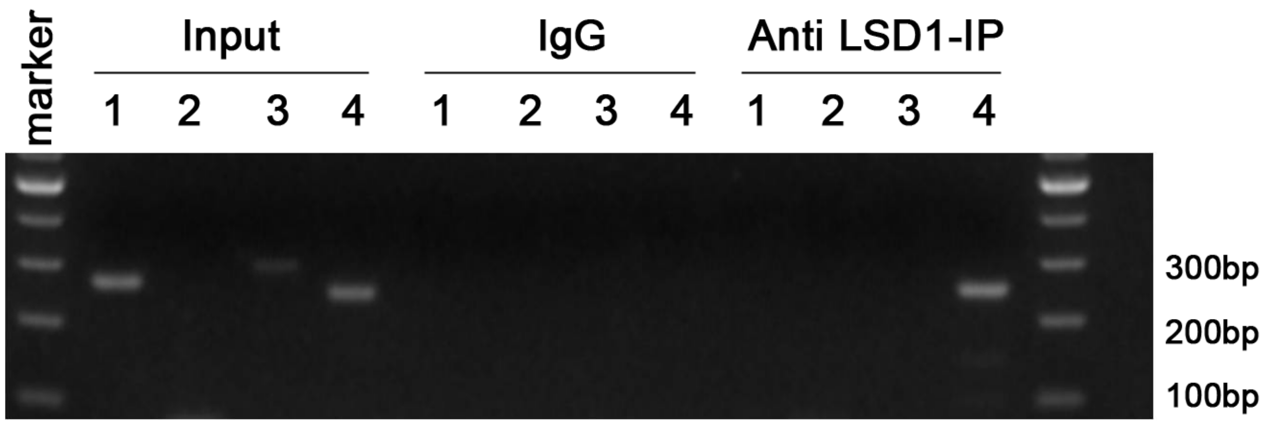

Supplementary Figure 4: Occupancy of LSD1 was detected specifically at the TSSs (transcription start sites) of Vimentin (No.4) in C33A cells line compared to three gene sequences within upstream of target genes in the range of 800bp (No.1, No.2, No.3).

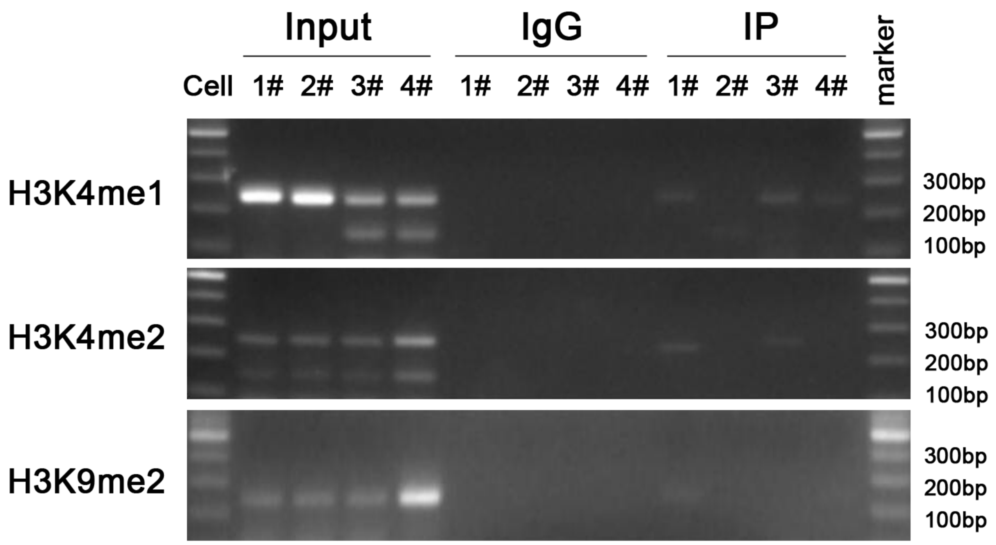

Supplementary Figure 5: RT-PCR analysis shows the enrichment of H3K4me1, H3K4me2, and H3K9me2 in Vimentin promoter. The 1#, 2#,3#,4#, four groups refer to the blank group, LSD1-overexpressed group, HPV16E7-overexpressed group and both LSD1 and HPV16 E7 over-expressed group, respectively.

Supplementary Table 1: The expression of LSD1 in cervical tissue types

Pathological type	Index of LSD1 expression			
	—	+	++	+++
Normal cervix	1	10	26	8
CIN	0	4	12	19
SCC	1	4	26	68
Other cervical carcinoma	0	3	8	3

Supplementary Table 2: The expression of LSD1 in different cervical tissue types

Pathological types	mean±s.d.
NC	5.6±2.3
CIN	6.3±2.0
SCC	7.9±1.9
Other cervical cancer	5.1±1.6

Supplementary Table 3: The expression of LSD1 in different cervical tissue types

The significant comparison of different groups	p value
NC vs SCC	<0.001
NC vs CIN	0.1416
CIN vs SCC	<0.001

Supplementary Table 4: The expression of LSD1 in normal cervical and cervical cancer tissues

Pathological type	LSD1					
	Sum	Negative	Positive			P value*
			+	++	+++	
SCC	28	0	3	10	15	0.0025
Adjacent normal tissue	28	0	8	19	1	
